# Transcriptomic Divergence and Associated Markers Between Genomic Lineages of Silver Catfish ( *Rhamdia quelen* )

**DOI:** 10.1002/ece3.71021

**Published:** 2025-03-13

**Authors:** Néstor Ríos, Belén G. Pardo, Carlos Fernández, José Antonio Alvarez‐Dios, Paulino Martínez, Carmen Bouza, Graciela García

**Affiliations:** ^1^ Sección Genética Evolutiva, Facultad de Ciencias UdelaR Montevideo Uruguay; ^2^ Departamento de Zoología, Genética y Antropología Física, Facultad de Veterinaria, Campus Terra Universidade de Santiago de Compostela Lugo Spain; ^3^ Departamento de Matemática Aplicada, Facultad de Matemáticas Universidade de Santiago de Compostela Santiago de Compostela Spain

**Keywords:** genomic lineages, local adaptation, transcriptomic divergence

## Abstract

*Rhamdia quelen* is a catfish widely distributed throughout South America, characterized by a complex taxonomic history. This species is a valuable resource for both fisheries and aquaculture. Due to its cultural and economic importance, it has been prioritized for conservation in the Neotropical region. Population genomics studies supported two main lineages latitudinally distributed (North and South) in the Neotropical basins Río de la Plata and Laguna Merín based on current genetic isolation and signals of local adaptation. In this study, we characterized the *R. quelen* transcriptome in brain, head kidney, liver, skeletal muscle, testis, and ovary by RNAseq to target genes and associated markers involved in key adaptive traits. After filtering, a comprehensive catalog of 24,433 transcripts was annotated, providing insights into the immune function of head kidney and liver, the association of brain with the endocrine system, and the metabolic function of liver. Skeletal muscle and brain expressed genes associated with growth were also identified. Transcriptomic differences suggestive of adaptation to temperature and salinity were revealed between North and South genomic lineages. A total of 100,045 SNPs loci were identified within transcripts, most of them (78.8%) showing low genetic differentiation between lineages (F_ST_ ≤ 0.100). However, 2504 loci (2.5%) showed high differentiation (F_ST_ ≥ 0.800), some of them located within genes associated with putative adaptation of genomic lineages to environmental factors such as temperature and salinity. These SNPs represent useful gene markers for future functional and population genomic studies for sustainable management of wild populations and their application in breeding programs.

## Graphical abstract

We present transcriptomic divergence between two latitudinally distributed lineages of *Rhamdia quelen* , which could explain local adaptations and the speciation processes proposed in previous studies. Moreover, the differentiation of transposable elements between lineages could also be associated with this speciation process.

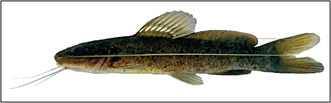

## 1 Introduction

**FIGURE 1:**
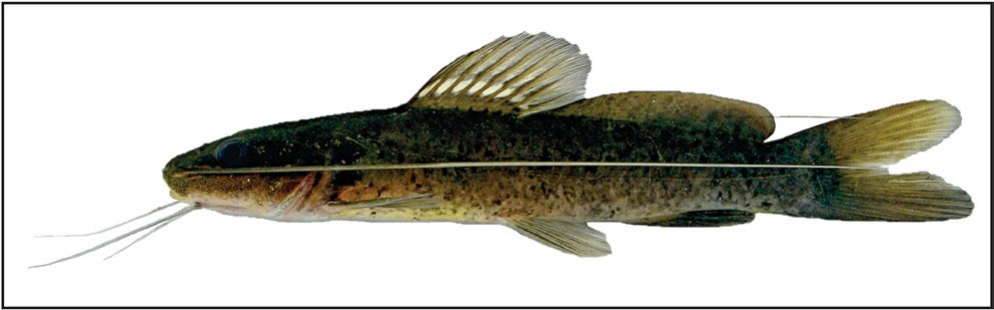
*Rhamdia quelen* specimen from the Río Uruguay basin. Photo by Wilson Sebastián Serra.

*Rhamdia quelen* (Figure 1), commonly known as the silver catfish, is widely distributed throughout South America, specifically from the Northeast of the Los Andes mountain range to the center of Argentina (Perdices et al. 2002; Ríos et al. 2017). This species is a valuable resource for fisheries and aquaculture due to its zootechnical and market quality (Pérez‐Atehortúa et al. 2022; Scaranto et al. 2018). It is cultivated in semi‐intensive and intensive systems and excavated ponds, as well as net cages, specifically in the temperate and subtropical zones of South America (Bombardelli et al. 2021; Scaranto et al. 2018). As a native species, it is adapted to thermal variation and exhibits optimal growth rates in southern Brazil, the La Pampa region of Argentina, and Uruguay (Lins Rodrigues et al. 2021; Mauerwerk et al. 2021; Santos and Meurer 2020). Due to its cultural and economic value, it has been prioritized for conservation in its southernmost distribution (Loureiro et al. 2013). Nevertheless, functional genomics information is lacking to understand its adaptation to a diverse environment.

**FIGURE 2:**
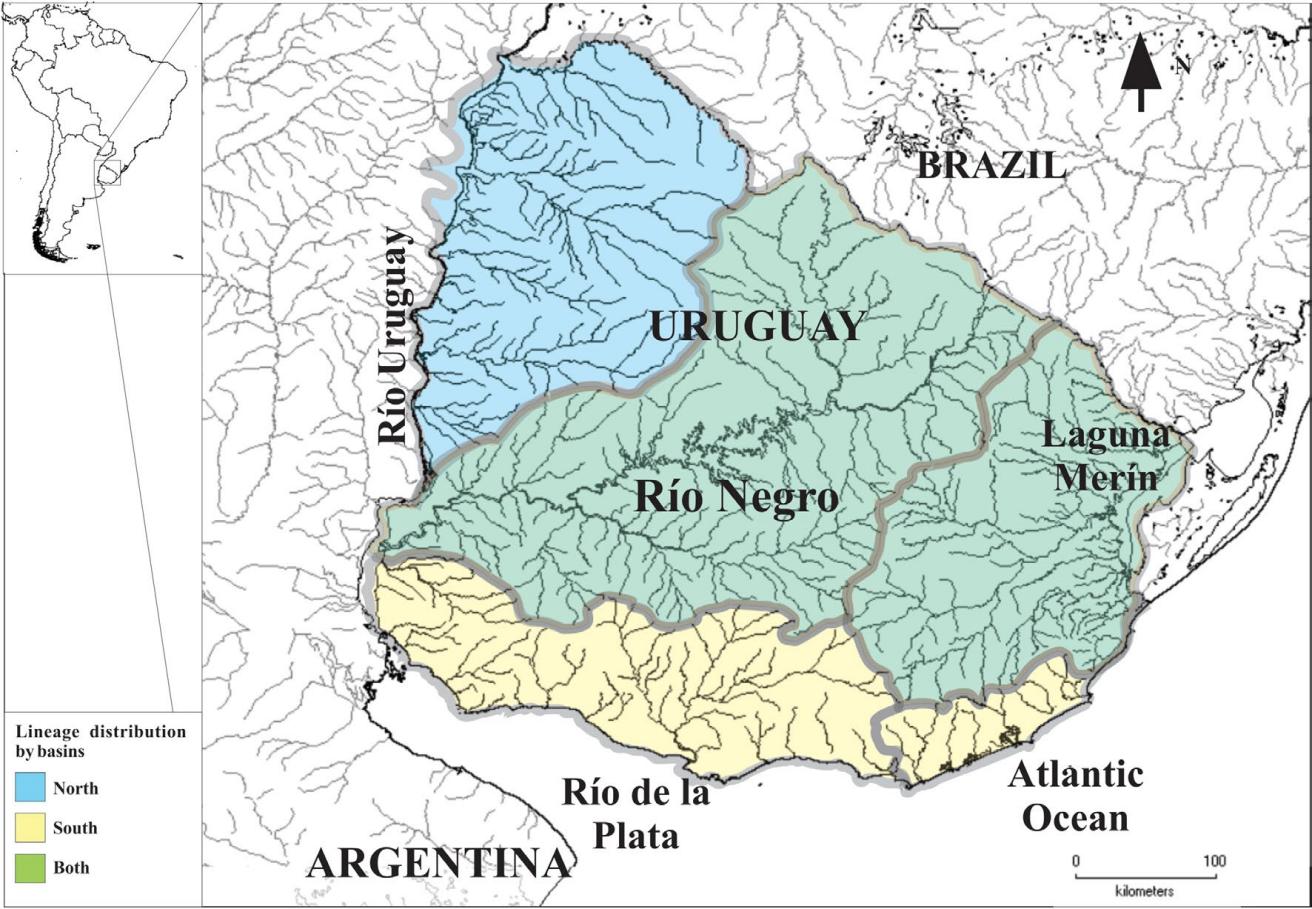
Collection sites of analyzed *Rhamdia quelen* samples from the Río Arapey, Río Uruguay basin, and Laguna Blanca, a coastal lagoon in the SW Atlantic Ocean basin. Distribution of North and South genomic lineage are highlighted in blue (Río Uruguay basin) and yellow (Río de la Plata and SW Atlantic Ocean basin), therefore, basins where they coexist are highlighted in green (Río Negro and Laguna Merín).

This species presents a complex taxonomic history, with 46 nominal species synonymized by Silfvergrip (1996). Subsequent molecular and morphological evidence reappraised its taxonomy (Garavello and Shibatta 2016; Hernández et al. 2015; Perdices et al. 2002). Studies using mitochondrial DNA (mtDNA) identified various lineages in South America (Ribolli et al. 2017; Ríos et al. 2017; Scaranto et al. 2018; Usso et al. 2019). Microsatellite data supported ancestral hybridization between at least two of these mitochondrial lineages (Rq4 and Rq6) (Ríos et al. 2018). Population genomics analysis, based on type IIB endonucleases Restriction site‐Associated DNA sequencing (2b‐RADseq) and de novo genotyping by sequencing, permitted the identification of two genomic lineages in the Río de la Plata and Laguna Merín major basins: the North lineage, including Río Uruguay, Río Negro, and Laguna Merín basins, and the South lineage comprehending Río Negro, Laguna Merín, and coastal lagoons (Laguna del Sauce, Laguna Blanca, Laguna de Rocha and Laguna Castillos) (Figure 2) (Ríos et al. 2020). Despite ancestral introgression, Rq4 and Rq6 mtDNA lineages were mostly associated with the North and South lineages, respectively, in accordance with no evidence of recent gene flow between these genomic lineages (Ríos et al. 2020). In fact, the pattern of genetic differentiation observed was apparently associated with temperature and latitude, suggesting local adaptation. Ríos et al. (2020) also found genomic regions putatively under selection that were associated with adaptation to salinity. In this evolutionary context, the development of the first transcriptomic resources and associated SNP markers would aid in providing functional evidence of the genomic divergence related to the mechanism of adaptation or phenotypic plasticity observed between lineages in *R. quelen* .

The term ‘transcriptome’ refers to the RNA molecules or transcripts, including protein‐coding messenger RNA (mRNA) and non‐coding RNA (ncRNA), expressed in a cell or tissue at a given time point under specific developmental or environmental conditions (Naumova et al. 2013; Waiho et al. 2022). The gene expression profile of each organ or tissue is closely associated with its specific functions (Liao et al. 2013; Mohamed et al. 2018), and therefore, it is crucial to investigate the transcriptome of key organs involved in adaptive processes. For instance, the brain plays a pivotal role in endocrine and homeostasis regulation and is fundamental for learning and behavior; further, it is involved in the growth and reproduction of fish (Chu et al. 2021; Liu et al. 2018; Robledo et al. 2017; Winberg and Thörnqvist 2016). The head kidney is an essential immune organ in fish (Xu et al. 2018), while the liver plays a principal role in digestion, energy metabolism, and protein biosynthesis, but also in some crucial immune functions (Qiao et al. 2016). Additionally, the liver is responsible for metabolic adjustments to stressors (Aluru and Vijayan 2009). Skeletal muscle, which comprises around half of fish body mass (Palstra et al. 2013; Valenzuela et al. 2018), plays a fundamental role in metabolic homeostasis and locomotion (Palstra et al. 2013; Valenzuela et al. 2018; Zhu et al. 2021). Although the ovary and testis are morphologically and functionally distinct sexual organs, they originate from the same gonadal primordium and are decisive for reproduction (Nishimura et al. 2016). The transcriptome of organs and tissues can evolve at varying rates and exhibit divergence between populations (El Taher et al. 2021). As a result, numerous studies have explored transcriptome differences between populations, strains or ecotypes (Köbis et al. 2013; Lenz et al. 2013; Schunter et al. 2014; García et al. 2019; Jeffries et al. 2019; Chu et al. 2021), providing functional genomic insights even in the absence of a reference genome (Chandhini and Kumar 2019). Transcriptomic profiles of individuals or populations enable understanding the genetic mechanisms underlying local adaptation or phenotypic plasticity (Liu et al. 2021; Ma et al. 2016). In the case of *R. quelen* , differential transcriptomics between genomic lineages might be associated with local adaptation to temperature and salinity, the main environmental factors in the region, as suggested by Ríos et al. (2020).

Our study aimed at characterizing the transcriptome of *R. quelen* , focusing on the brain, head kidney, liver, skeletal muscle, testis, and ovary as major organs related to essential functions of fish potentially involved in adaptive processes. Furthermore, we explored the transcriptome differences between two *R. quelen* genomic lineages to unveil functional divergence associated with previously suggested local adaptation.

## 2 Materials & Methods

### 2.1 Sample Collection and mtDNA Lineage Characterization

A total of 10 *R. quelen* adult specimens of the South lineage (P2315–P2324) and 13 of the North lineage (P2301–P2313) were collected in the summer of 2018 from Laguna Blanca and Río Arapey, respectively (Figure 2). This period falls within the reproductive season of *R. quelen* . Sampling was conducted during the night and the morning. All specimens processed for transcriptome analysis were adults exceeding a total length of 20 cm, and their sexes were recorded (Data S1). Samples of brain, head kidney, liver, skeletal muscle (lateral posterior), testis, and ovary were collected and preserved in RNAlater in the field, stored at 4°C overnight, and then transferred to −80°C until RNA extraction. Only pre‐spawning gonads were considered for transcriptome analysis. Additionally, for DNA extraction, a liver sample from each individual was stored in 95% ethanol.

Identification of mtDNA lineage was conducted using the cytochrome b marker following Ríos et al. (2020). Only individuals from Laguna Blanca belonging to the Rq6 mtDNA lineage were considered as the South lineage. Similarly, specimens from Río Arapey with the Rq4 mtDNA lineage were exclusively classified as the North genomic lineage. All sampling protocols were approved by the CNEA (Comisión Nacional de Experimentación Animal) from Uruguay.

### 2.2 RNA Extraction, Library Construction and Sequencing

Total RNA from brain (B), liver (L), ovary (OVA) and testis (TES) was isolated using the RNeasy Mini Kit (Qiagen, 74,104). For skeletal muscle (M) and head kidney (K), total RNA extraction was carried out using the Direct‐zol RNA Miniprep Kit (R2050) and TRI Reagent due to the higher performance of these protocols on these organs. The quantity and quality of total RNA were assessed using a NanoDrop ND‐1000 spectrophotometer (NanoDrop Technologies Inc) and agarose gel (2%) electrophoresis, respectively. Subsequently, RNA from 22 different individuals was pooled to construct 10 libraries (Data S1): the brain of the North (B‐N) and South (B‐S) lineages; the head kidney of the North (K‐N) and South (K‐S); the liver of the North (L‐N) and South (L‐S); the skeletal muscle of the North (M‐N) and South (M‐S); the ovary (OVA); and the testis (TES). B‐N, K‐N, L‐N and M‐N pools were constructed using groups of 10 different individuals due to the amount of available RNA (Data S1), while B‐S, K‐S, L‐S and M‐S pools were created using the same 10 specimens (Data S1). Ovary and testis transcriptomes were not included in the lineage comparison because the OVA and TES pools were constructed with only four females (two from the South and two from the North lineages) and two males (only from the South lineage, since no male gonads from the North lineage could be sampled) (Data S1). Each pool was constructed with equal RNA amount of the samples. RNA quality of the pools was assessed using a 2100 Bioanalyzer (Agilent Technologies). Subsequently, the 10 cDNA libraries were constructed using the TruSeq Stranded mRNA Library Prep Kit (Illumina, USA) and sequenced on an Illumina Hiseq4000 with a 100‐bp paired‐end (PE) read strategy by Macrogen (Korea).

### 2.3 Read Filtering, De Novo Transcriptome Assembly and Functional Annotation

Read quality was assessed using FastQC (http://www.bioinformatics.babraham.ac.uk/projects/fastqc/; version 0.10.1). A filtering step for leading and trailing bases with a Phred score ≥ Q20 was applied and reads with a 4‐base sliding window average Phred score below 20 were discarded. Pair‐end (PE) reads were processed to eliminate residual Illumina adapters using Trimmomatic‐0.38 (Bolger et al. 2014). Only PE reads longer than 36 bp were retained. Given the absence of a reference genome or transcriptome for *R. quelen* , PE reads from the 10 libraries were de novo assembled using Trinity v. 2.6.6 (Grabherr et al. 2011) with default options. The Trinity assembly was further clustered using CAP3 (Huang and Madan 1999). Transcript annotation was performed using BLASTx (Basic Local Alignment Search Tool; Altschul et al. 1990) searches against the NCBI (National Center for Biotechnology Information) non‐redundant (nr) protein database and UniProt Reference Clusters 90 (Bateman et al. 2021), with a cut‐off E‐value of 10^−5^. To ensure accurate annotation, if the first annotation resulted in ‘hypothetical’ or ‘uncharacterized’ an alternative annotation was selected. Only annotations with homology to a fish taxon were considered. The annotated sequences were filtered to reduce redundancy using the EvidentialGene tr2aacds pipeline (Gilbert 2013). Redundant sequences with > 99% homology and > 30% coverage using BLASTn against the same annotated sequences were removed. Finally, only one Trinity singlet was retained for each Trinity cluster.

Gene Ontology (GO) annotation was performed in BLAST2GO (Conesa et al. 2005) with BLASTn (E‐value cut‐off 10^−5^). Two annotations based on KEGG (Kyoto Encyclopedia of Genes and Genomes) orthology were performed by two methodologies. The KEGG Automatic Annotation Server (Moriya et al. 2007) was applied to identify KEGG objects using reference genomes of several fish (i.e., *Ictalurus punctatus* , *Pangasianodon hypophthalmus*, *Danio rerio* , *Takifugu rubripes* , *Oreochromis niloticus* , *Oryzias latipes* , *Cyprinus carpio* and *Latimeria chalumnae* ) and *Homo sapiens. Homo sapiens* was used for its more extensive annotation available as compared with fish (Huff et al. 2018). On the other hand, KOBAS (KEGG Orthology Based Annotation System) (Bu et al. 2021) local annotation was conducted against the *D. rerio* database for enrichment analyses based on KEGG orthology.

### 2.4 Transcriptome Characterization

The paired trimmed reads from different samples were aligned to the de novo transcriptome and expression was quantified with Kallisto ver. 0.46.1 (Bray et al. 2016) using a threshold of one transcript per million (TPM) to be retained. An UpSet plot built in ChiPlot (https://www.chiplot.online, accessed on 24 November 2024) was used to compare expressed transcripts among different organs (combining samples from both lineages). GO term enrichment analyses were performed using OmicsBox ver. 2.0.8 (BioBam Bioinformatics 2019). KEGG pathway enrichment analyses were performed with *KOBAS*. The *R. quelen* transcriptome generated in this study was used as a reference background for GO enrichment, while the *D. rerio* database was used for KEGG pathway enrichment. Fisher's exact test and Benjamini‐Hochberg correction for multiple testing (False Discovery Rate (FDR) < 0.05) were applied for enrichment analyses. Finally, we screened the *R. quelen* transcriptome by applying RepeatMasker version 4.1.5 (Smit et al. 2013) on FishTEDB, a fish database of transposable elements (TE) (Shao et al. 2018), to characterize TEs genes expressed in the two lineages (considering B, K, L and M) and the whole transcriptome.

### 2.5 Single Nucleotide Polymorphism (SNP) Calling and Analysis

Polymorphism in coding expressed sequences was assessed using SAMtools v.1.8 (Li et al. 2009) and PoPoolation v.2.1201 (Kofler et al. 2011) to identify SNPs associated with transcripts in *R. quelen* . The analysis employed a minimum depth of 400 reads per locus and a minimum allele count (MAC) of 20. This analysis involved concatenating B‐N, K‐N, L‐N, and M‐N reads for the North lineage, and B‐S, K‐S, L‐S, and M‐S reads for the South lineage, to identify SNP variants at the population level. The F_ST_ statistic per locus to assess genetic differentiation between lineages was estimated using the PoPoolation program. The RNAseq SNPs detected in this study were crossed with our previous 2b‐RADseq dataset (Ríos et al. 2020). These 2b‐RADseq data were reanalyzed using as reference the transcriptome here assembled, while Ríos et al. (2020) performed a de novo 2b‐RADseq analysis. The previous 2b‐RADseq dataset included 71 specimens from nine localities in riverine and coastal lagoon basins. In the reanalysis, the new 2b‐RADseq genotypes were called using STACKS ver. 2.62, aligning the fastq files against the assembled transcriptome using BWA‐MEM v. 0.7.12 (Li 2013). The genotypes per locus and F_ST_ between lineages derived from RNAseq SNPs were contrasted with those updated RADseq SNPs using Genepop ver. 4.7.0 (Rousset 2008).

## 3 Results

### 3.1 Transcriptome Assembly and Functional Annotation

The entire transcriptomic information of the analyzed samples was used to reconstruct the first *R. quelen* transcriptome based on RNAseq over 345 M total raw PE reads (average 34.56 M PE reads per pool; Data S2; NCBI Short Read Archive database: SRA accession number PRJNA1091164). Approximately 94% of reads were retained after filtering (Data S2). Among libraries, quality was similar (Q20%‐Q30% and filtered reads), although the RNA Integrity Number (RIN) was variable (Data S2). The B‐S and OVA RIN values were rather low, which may bias the results for gene expression analysis, but scarcely influencing transcriptome reconstruction. The de novo assembly using Trinity resulted in 368,919 unique transcripts that were further reduced after CAP3 clustering to 23,615 contigs and 296,019 singletons. A BLASTx similarity search (*E*‐value < 10^−5^) detected significant homology for 128,345 transcripts (40.15%) and 130,278 (40.76%) with the NCBI non‐redundant (nr) protein database and UniProt Reference Clusters 90, respectively. After annotation and homology filtering (see M&M), 24,837 annotated transcripts were retained. Finally, 404 transcripts were removed by the NCBI contamination screen, resulting in a final transcriptome of 24,433 transcripts (Genbank: GKTU00000000; Data S3). The length of these transcripts ranged between 201 and 30,789 bp and the N50 value of the final assembly was 2863. Based on sequence similarity, one or more GO terms were retrieved for 8917 transcripts (36.50%). A total of 25,857 GO terms were identified, of which 9783 corresponded to biological process (BP), 10,563 to molecular function (MF), and 7,511 to cellular component (CC) categories (Data S3). Table 1 shows the top 10 GO terms according to BP, MF, and CC categories. The same information is shown for KEGG pathways in Table 1 and Data S3.

**TABLE 1:** Top ten GO terms and KEGG pathways of the *Rhamdia quelen* transcriptome.

Cell component	Amount	Biological process	Amount
Integral component of membrane	2360	DNA integration	295
Nucleus	815	Regulation of transcription DNA‐templated	281
Membrane	587	Protein phosphorylation	276
Cytoplasm	436	Proteolysis	256
Extracellular region	325	Signal transduction	246
Plasma membrane	325	RNA‐dependent DNA biosynthetic process	232
Extracellular space	112	G protein‐coupled receptor signaling pathway	187
Ribosome	89	Transmembrane transport	176
Endoplasmic reticulum membrane	87	Regulation of transcription by RNA polymerase II	149
Integral component of plasma membrane	74	Transposition, DNA‐mediated	145
Total cellular component	7511	Total biological process	9783

Molecular function	Amount	KEGG pathways	Amount
ATP binding	706	Metabolic pathway	1006
DNA binding	541	Neuroactive ligand‐receptor interaction	251
Metal ion binding	517	Phagosome	216
Nucleic acid binding	410	MAPK signaling pathway	206
Zinc ion binding	362	Endocytosis	193
Calcium ion binding	334	Calcium signaling pathway	179
GTP binding	238	Regulation of actin cytoskeleton	175
RNA‐directed DNA polymerase activity	232	Cellular senescence	146
DNA‐binding transcription factor activity	183	Focal adhesion	133
RNA binding	183	Vascular smooth muscle contraction	132
Total molecular function	10,563	Pathways identified in all transcripts	11,450

### 3.2 Transcripts Expressed by Tissues and Organs

**FIGURE 3:**
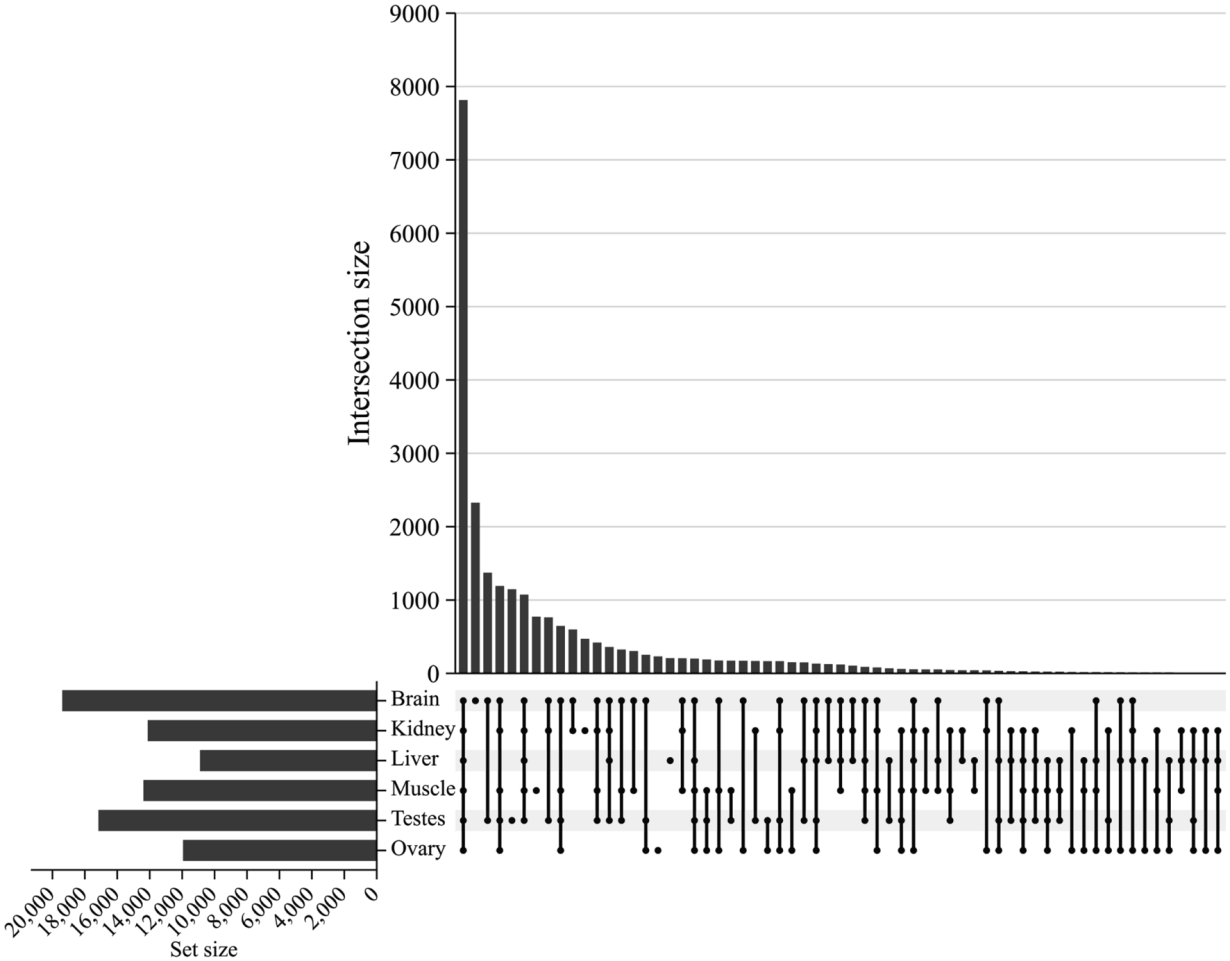
Comparison of expressed transcripts among brain, liver, head kidney, skeletal muscle, ovary, and testicle represented in an UpSet plot. Data S3 provides details of expressed transcripts by organ or tissue and describes expressed transcripts exclusively in one organ or tissue, expressed transcripts shared by all organs and tissues, and expressed transcripts shared by the two reproductive organs.

Data S3 and Figure 3 show the total transcripts identified and those expressed per organ (brain, head kidney, liver and skeletal muscle) combining both lineages. Data S3 also shows the transcripts expressed per pool (B‐N, B‐S, K‐N, K‐S, L‐N, L‐S, M‐N, M‐S, TES and OVA). The brain transcriptome was the most diverse (19,389 transcripts), followed by testis (17,161), skeletal muscle (14,383), head kidney (14,119), ovary (11,941) and liver (10,897). A total of 7815 transcripts were expressed in all six organs (Figure 3; Data S4). Additionally, 2326 transcripts were expressed exclusively in the brain, 471 in the head kidney, 207 in the liver, 772 in skeletal muscle, 231 in the ovary, and 1,146 in the testis. Exclusive transcripts in the brain were associated with the endocrine system, for example, parathyroid hormone (PTH) 2, thyrotropin‐releasing hormone receptor, and growth hormone‐releasing hormone receptor a. The head kidney was tightly associated with the immune system, for example, interferon‐induced very large GTPase 1, C‐C chemokine receptor type 5, and numerous transcripts annotated as immunoglobulins. Liver transcripts were associated with digestive processes; skeletal muscle with growth and fiber development, and contraction; while ovary and testis transcripts were associated with gonad development and differentiation, including doublesex‐ and mab‐3‐related transcription factors (*dmrt*), as well as *foxl2*, *sox9*, *cyp19a1*, *wnt4*, *stra8*, and *sox3*.

The GO term enrichment by organ is shown in Data S5. Brain transcripts were enriched in biological processes associated with signaling, cellular communication, and regulation of biological and cell process; head kidney showed a predominance of metabolic processes, but also in immune system processes and response to stress; liver was associated with cellular metabolic, metabolic processes, and response to stress; skeletal muscle with cellular metabolic processes and several biosynthetic processes; and finally, ovary and testis with lipid metabolism and chromosome processes. Several KEGG pathways were enriched in all organs, including metabolic pathways, endocytosis, herpes simplex virus 1 infection, RNA transport, cellular senescence, and focal adhesion (Data S5).

### 3.3 Comparative Transcriptomics in *R. quelen*

A total of 22,058 transcripts were expressed in at least one of the lineages across the organs analyzed: brain, head kidney, liver, and skeletal muscle, excluding testis and ovary (Table 2; Data S6). The majority (17,904) were shared between lineages, whereas 2366 and 1787 were exclusive to the South and North lineages, respectively (Table 2; Data S6). The brain (North vs. South: 7.9% vs. 12.3%) and the liver (0% vs. 5.1%) showed a higher number of exclusive transcripts in the South lineage, while the opposite was observed in the head kidney (12.4% vs. 7.1%) and skeletal muscle (16.6% vs. 13.9%), where a higher number of transcripts were exclusive to the North lineage. GO terms and KEGG pathways enrichment of exclusive transcripts from the North and South lineages are detailed in Data S6.

**TABLE 2:** Comparison of expressed transcripts between lineages, considering the four sampled organs collectively and individually by organ: Brain, head kidney, liver, and skeletal muscle.

	Four organs	Brain	Head Kidney	Liver	Skeletal muscle
Shared	17,904	15,462	11,372	10,344	9991
North unique	1787	1536	1746	0	2387
South unique	2366	2391	1001	553	2005
Total	22,057	19,389	14,119	10,897	14,383

Among consistent transcripts (TPM ≥ 5) detected in only one lineage, relevant coding genes found in the North lineage were related to the immunological response, such as toll‐like receptor 1, macrophage mannose receptor‐1‐like, Ig‐like domain‐containing proteins, C1q domain‐containing protein, and H‐2 class I histocompatibility antigen. Among those transcripts only detected in the South lineage also immunological genes were also found, including interferon regulatory factor 1‐like, galectin‐3‐like, claudin‐10, and cytokine receptor family member B12. Different ion transport‐associated genes were detected exclusively in one lineage (North: voltage‐dependent L‐type calcium channel subunit alpha‐1D‐like and Na^+^‐dependent phosphate cotransporter; South: potassium voltage‐gated channel subfamily H member 5a).

Several transcripts annotated as TEs were detected in only one lineage, particularly retrotransposons (Data S6), although TEs were found with similar frequency in the transcriptomic data of both lineages (Data S7), both for retroelements (North lineage: 9.99%; South lineage: 10.07%) and DNA TEs (North: 3.68%; South: 3.62%). Among retroelements, LINEs were more common than SINEs and LTR elements. Most TEs in FishTEDB were detected in both lineages (L2/CR1/Rex, R1/LOA/Jockey, R2/R4/NeSL, RTE/Bov‐B, L1/CIN4, BEL/Pao, Ty1/Copia, Gypsy/DIRS1 and Retroviral). CRE/SLACS, Penelope, and En‐Spm were not detected in the transcriptomic data from this study (Data S7).

### 3.4 Single Nucleotide Polymorphism Identification and Analysis

SAMtools and PoPoolation analyses identified 100,045 SNPs within 2414 protein‐annotated transcripts (Data S8). A total of 90,724 were biallelic, representing 66,247 transitions and 24,477 transversions (Ts/Tv = 2.71). Among these, 1194 SNPs belonged to mtDNA (Contig 5456). A total of 2524 SNPs showed high F_ST_ values (> 0,8) when comparing North and South lineages (158 SNPs, F_ST_ = 1.000; 1519, F_ST_ ≥ 0.900; and 2524, F_ST_ ≥ 0.800; Data S8). Loci with F_ST_ above 0.800 showed a lower Ts/Tv ratio (1.75) than the average (2.71). As might be expected for divergent mitochondrial lineages, a high number of loci with F_ST_ ≥ 0.800 (365 loci) were found in mtDNA. Among nuclear genes, chondroitin sulphate proteoglycan 5b showed several alleles fixed for alternative variants in the North and South lineages (F_ST_ = 1.000). Additionally, several relevant genes associated with the immune system showed high genetic differentiation (F_ST_ > 0.800). For instance, the Sodium/potassium‐transporting ATPase subunits alpha gene showed the highest number of highly divergent SNPs in the nuclear genome; four transcripts annotated as heat shock protein (*hsp*) showed 10 SNPs with F_ST_ ≥ 0.800, as did the insulin‐like growth factor binding protein 2b; finally, transcription factors, including transcription initiation factor tfIId subunit 7, hypoxia‐inducible factor 1‐alpha, and Krueppel‐like factor, included several SNPs with F_ST_ > 0.800.

A total of 7719 SNPs were identified in the reanalysis of the 2b‐RADseq dataset from Laguna Blanca and Río Arapey using the assembled transcriptome as reference. Out of these, 147 2b‐RADseq SNPs matched with those identified from RNAseq in this study (Data S9). The alleles identified in both analyses (new 2b‐RADseq vs RNAseq) were mostly consistent, and only four SNPs showed discordant information (Data S9). Among the 143 SNPs, the F_ST_ estimation was mostly consistent between 2b‐RADseq and RNAseq data considering the different data sources (average difference: 0.109; range: 0.000–0.728; Data S9).

## 4 Discussion

Here we report the first transcriptome assembly of *R. quelen* , a valuable Neotropical genetic resource for fisheries and aquaculture, covering samples from two divergent genomic lineages to provide functional information to support the signs of adaptive variation reported (Ríos et al. 2020) through a transcriptional approach.

The de novo transcriptome assembly resulted in the characterization of 24,433 transcripts expressed in various organs of *R. quelen* , including the brain, head kidney, liver, skeletal muscle, testis, and ovary. Other de novo transcriptomes in related fish species have reported a higher number of annotated transcripts; for example, 163,949 transcripts and 89,579 genes in the muscle of lanzhou catfish ( *Silurus lanzhouensis* , Siluriformes; Xiao et al. 2022); 42,122 transcripts in the liver of yellow catfish ( *Pelteobagrus fulvidraco* ; Siluriformes; Liu et al. 2017), and 60,477 transcripts in the gonad of amur catfish (*Silurus asotus*; Siluriformes; Shen et al. 2020). Beyond differences in sampling, sequencing, and assembly approaches among studies, these differences may also be attributed to our strict filtering criteria to avoid redundancy and the inclusion of only annotated transcripts for the final transcriptome.

### 4.1 Characterization of Organ Transcriptomes

The brain exhibited the highest transcriptome diversity (19,389 transcripts), consistent with previous studies in vertebrates (Lai et al. 2015; Naumova et al. 2013). The brain of *R. quelen* showed several exclusive transcripts associated with the endocrine system and was enriched in gonadotropin‐releasing hormone signaling pathways. Genes associated with the endocrine system, especially those related to gonadotropins, are of particular importance for the reproduction of aquaculture species and can be used for improving gamete quality in breeding programs (Triantaphyllopoulos et al. 2019). As well known, the fish head kidney and liver play crucial roles in the immune system (Xu et al. 2018). Both organs were enriched in nucleotide‐binding and oligomerization domain‐like receptor signaling pathways, and the head kidney was enriched in C‐type lectin receptor signaling pathway. Additionally, the head kidney revealed exclusive transcripts associated with immune response, while the metabolic function of the liver (Qiao et al. 2016) was evidenced by enriched metabolic pathways (e.g., carbon metabolism, glycerophospholipid metabolism, pyrimidine metabolism, glycolysis/gluconeogenesis and biosynthesis of amino acids). The skeletal muscle transcriptome was enriched in transcripts related to cellular metabolism (cellular, protein, nucleic acid, macromolecule catabolic, lipid and phosphorus metabolic processes), reflecting its locomotor role and energetic demand (Palstra et al. 2013). The muscle transcriptome also showed enrichment in several biosynthesis processes and the exclusive expression of growth hormone‐regulated Tre2‐Bub2‐Cdc16, associated with muscle growth and energy requirements (Mommsen 2004; Robledo et al. 2016). Gonadal transcriptomes were enriched in metabolism and biosynthesis‐related functions, reflecting the energy cost of gamete production during the reproductive season (Carnevali et al. 2017; do Carmo Silva et al. 2019). Both gonads were enriched in several lipid metabolic processes. Previous studies have suggested that lipids would facilitate gonad development (Leng et al. 2019; Zhou et al. 2022). The presence of multiple *dmrt* family copies in both gonads is a significant finding, particularly given the elusive nature of sexual determination in *R. quelen* (Fernandino and Hattori 2019) and could be applied for monitoring reproductive management in aquaculture for this species. This is a key issue that should be further explored using the transcriptomic data from this study.

### 4.2 Analysis of Transcriptomic Differentiation Between *R. quelen* Lineages

This study allowed uncovering gene expression profiles putatively involved in adaptation to different environments of *R. quelen* lineages (Ríos et al. 2020) to be explored as functional biomarkers for population transcriptomic and evolutionary studies (Tamagawa et al. 2022). Over 4000 transcripts were exclusively detected in only one lineage in the samples studied: 2366 and 1787 in the South and the North lineages, respectively. The transcriptome differentiation between lineages may be explained by the divergence of orthologous genes after isolation, by the expression of different paralogous genes within each lineage, or by phenotypic plasticity. Among them, specific subsets of transcripts associated with the immunological response were identified in each lineage, suggesting potential adaptations or response driven by exposure to different pathogens or environmental conditions, influenced by abiotic factors such as salinity or temperature (Makrinos and Bowden 2016). For instance, toll‐like and mannose receptor genes, expressed only by the North lineage, are responsible for recognizing different pathogens during immune responses (Dong et al. 2016; Rebl et al. 2010). Similarly, interferon regulatory factor and galectin‐3, expressed in the South lineage, are associated with immune responses against virus and bacteria, respectively (Sirisena et al. 2020; Tian et al. 2021).

Both lineages demonstrated similar representation of TE classes in the transcriptome; however, some abundant TE transcripts were lineage‐specific, consistent with the genomic differentiation observed and the specific dynamics of some TEs. *Rhamdia quelen* exhibited a notable abundance of expressed TEs in the assembled transcriptome in this study (15.66%), similar to *Anguilla marmorata* (range: 14.84%–18.02% when considering the variation among fresh, brackish and sea water), but higher than the cyprinids *Leuciscus waleckii* (1.6%, Xu et al. 2013) and *Ctenopharyngodon idella* (10.43%; Duan et al. 2022) from a 12‐organ transcriptome. On the other hand, *R. quelen* TE expression was lower than that reported for *Oryzias melastigma* (Beloniformes) and *Oncorhynchus keta* (Salmoniformes), where TE‐expressed variation among fresh, brackish, and sea water ranged between 23.16%–25.08% and 28.35%–29.26%, respectively (Carotti et al. 2022).

Interestingly, LINE retroelements were more abundant in the *R. quelen* transcriptome dataset of this study than in other transcriptome studies in teleost, where higher abundance of DNA transposons has been reported (Serrato‐Capuchina and Matute 2018). The predominance of LINEs observed in *R. quelen* also contrasts with that observed in other catfish genomes, such as *I. punctatus* and *P. hypophthalmus* , where DNA transposons also dominate. These results should be interpreted with caution because the TE genome distribution can differ between the coding regions and the whole genome (Lanciano and Cristofari 2020; Vera et al. 2015). For instance, TE transcripts could be expressed using a promoter that overlaps with other genes, and LINE and SINE transcripts could be overestimated due to the presence of a polyA sequence in canonical gene sequences (Lanciano and Cristofari 2020). TEs might play an important role in gene regulation (Gebrie 2023) and TE activity in eukaryotes has also been associated with speciation through genome rearrangement and differential gene expression (Ricci et al. 2018; Serrato‐Capuchina and Matute 2018). In this sense, further research about the putative role of TEs in the speciation process proposed by Ríos et al. (2020) will be necessary. The presence of lineage‐specific TE transcripts and the dominance of LINEs over DNA transposons in the transcriptomic data of both lineages, as well as the possible TEs overlap with differentially expressed genes between lineages, represent suggestive information on that purpose.

### 4.3 SNP Variants Associated With Lineage Divergence

We uncovered 100,045 SNPs within transcripts in *R. quelen*, a substantially higher number than the 17,559 previously obtained through 2b‐RADseq, mostly anonymous (Ríos et al. 2020). The Ts/Tv ratio serves as an indicator of selection constraints (Chandhini and Kumar 2019), showing variation across the genome; while the human rate is 3.0 for coding DNA, it is approximately 2.0 in the rest of the genome (Bainbridge et al. 2011; Yauy et al. 2023). In the case of *R. quelen* , the Ts/Tv ratio calculated for the transcriptome SNPs was 2.71. In humans, a Ts/Tv between 2.8 and 3.0 is considered a high‐quality exome variant, associated with consistent genotyping (DePristo et al. 2011; Yauy et al. 2023). In this sense, the Ts/Tv ratio of validated loci (2.84) was even higher than the total transcriptomic loci of *R. quelen* (2.71). The transcriptomic SNP Ts/Tv ratio (2.84) was higher than the rate based on the 17,559 genomic loci genotyped by 2b‐RADseq (2.25) (Ríos et al. 2020). It is expected that most loci identified through 2b‐RADseq are intergenic, so the differences might be explained by selective constraints on coding regions. SNPs with high F_ST_ values (≥ 0.800) exhibited a noticeably lower Ts/Tv rate (1.75), indicating a potential deviation due to divergent selection between North and South lineages, as previously suggested by Ríos et al. (2020). We cannot exclude that differentiation could be due to genetic drift acting on the two allopatric populations. The average F_ST_ over loci between the two lineages was 0.095, with most loci (78,811, 78.8%) showing F_ST_ values below 0.100. These F_ST_ values between the Río Arapey and Laguna Blanca populations were lower on average than those estimated by Ríos et al. (2020) between the same localities (F_ST_ = 0.496 based on 17,559 SNPs). As for Ts/Tv ratios, the differences observed in F_ST_ values could be attributed to selective constraints on coding regions (Josephs et al. 2015) with respect to anonymous 2b‐RADseq loci analyzed by Ríos et al. (2020). Among the loci with higher F_ST_ between North and South lineages, we identified 2524 loci (2.5%) with a F_ST_ > 0.800 (average: 0.922) higher than the 73 outlier loci (0.4%) with an F_ST_ > 0.910 reported by Ríos et al. (2020), supporting again a stronger effect of divergent selection on coding regions.

Among loci with higher F_ST_, some functional candidates may be pinpointed, such as 10 SNPs belonging to four genes annotated as heat shock proteins (*hsp*), highly conserved genes which play a role in the response to biotic and abiotic stressors (Basu et al. 2002; Mohanty et al. 2018). Differential expression of *hsp* genes, variation in the number of gene copies, or non‐synonymous changes in these genes could contribute to adaptation to new environmental factors (Chen et al. 2018), such as temperature differences, as reported by Ríos et al. (2020). Previous genetic studies on redband trout ( *Oncorhynchus mykiss gairdneri* ) found an association between thermal response and *hsp* genes, with a specific SNP in *hsp47* exhibiting a strong response to environmental adaptation (Narum et al. 2013). Additionally, 9 SNPs in the chondroitin sulphate proteoglycan 5b gene were fixed for alternative variants in both genomic lineages. Interestingly, chondroitin sulphate proteoglycan 5a has previously been associated with the adaptation of *Nemadactylus macropterus* (Centrarchiformes) to water temperature (Papa et al. 2022). We also identified several sodium/potassium‐exchanging ATPase complex genes that play a fundamental role in osmoregulation and ion exchange (Sebastian et al. 2018). Previous studies demonstrated the association between differential expression of this complex and adaptation to hypersaline or freshwater environments (Sebastian et al. 2018). Our results support the adaptation to saline environments, such as coastal lagoons in Uruguay that have direct or indirect connections to the Atlantic Ocean (e.g., Laguna Blanca, Laguna Rocha and Laguna de Castillos coastal lagoons). It is worth noting that even though Laguna Blanca currently lacks a direct connection to the Atlantic Ocean, it exhibits higher salinity compared to Río Arapey and other localities inhabited by the North lineage (Ríos et al. 2020).

The observed functional divergence between the two genomic lineages of *R. quelen* and the potential evidence of local adaptations should be taken into account when selecting founder specimens for aquaculture breeding programs. Specifically, considering the lack of evidence of hybrids between the two lineages in the wild (Ríos et al. 2020), it would be important to evaluate their reproductive success. Moreover, the deep genetic differentiation highlights the potential consequences of escapees from aquaculture farms, particularly artificial hybrids (Scaranto et al. 2018), as well as the translocation of individuals among different basins. Finally, the transcriptomic SNPs related to the immune system, stress tolerance, osmoregulation, and larval development could be valuable for further functional and association studies on adaptive variation (e.g., Andersen et al. 2023) and genetic breeding programs assisted by molecular markers.

## 5 Conclusions

We characterized the first *R. quelen* transcriptome from the brain, head kidney, liver, skeletal muscle, ovary, and testis, providing basic functional information for *R. quelen* on different key organs. Gene expression profiles were associated with the endocrine function of the brain, the immunological role of the head kidney and liver, the metabolism of the liver and skeletal muscle, and gonadal development related to key productive traits. This information is crucial to understand functional evolution and divergence between the North and South genomic lineages of *R. quelen* in Río de la Plata and Laguna Merín Neotropical basins. Indeed, we detected significant transcriptomic differences in genes related to stress and the immune system. Furthermore, the observed differences in TEs associated with transcripts that would deserve further studies, as they could play a special role in genomic divergence or speciation. We also explored allelic divergence of gene‐associated SNPs between lineages and detected a large repertory of SNP variants putatively associated with environmental adaptations, such as chondroitin sulfate proteoglycan 5, *hsp*, and the sodium/potassium‐exchanging ATPase complex. The identification of transcripts of interest and numerous SNPs holds significant potential for sustainable management of fisheries resources and expediting future genetic breeding programs in this taxon.

## Author Contributions

**Néstor Ríos:** conceptualization (equal), data curation (equal), formal analysis (equal), funding acquisition (equal), investigation (equal), methodology (equal), project administration (equal), writing – original draft (equal), writing – review and editing (equal). **Belén G. Pardo:** conceptualization (equal), funding acquisition (equal), investigation (equal), methodology (equal), visualization (equal), writing – original draft (equal), writing – review and editing (equal). **Carlos Fernández:** data curation (equal), formal analysis (equal), writing – review and editing (equal). **José Antonio Alvarez‐Dios:** data curation (equal), formal analysis (equal). **Paulino Martínez:** funding acquisition (equal), writing – original draft (equal), writing – review and editing (equal). **Carmen Bouza:** conceptualization (equal), funding acquisition (equal), investigation (equal), methodology (equal), visualization (equal), writing – original draft (equal), writing – review and editing (equal). **Graciela García:** conceptualization (equal), funding acquisition (equal), supervision (equal), writing – original draft (equal), writing – review and editing (equal).

## Conflicts of Interest

The authors declare no conflicts of interest.

## Supporting information


Data S1.

Data S2.

Data S3.

Data S4.

Data S5.

Data S6.

Data S7.

Data S8.

Data S9.

## Data Availability

Genetic data: Raw sequence reads are deposited in the SRA (BioProject PRJNA1091164). Final transcriptome of 24,433 sequences is deposited in Genbank: GKTU00000000. Mitochondrial cytochrome b sequences are deposited in Genbank: PP898343 –PP898366. Sample metadata: Metadata are also stored in the SRA (BioProject PRJNA1091164).
