# Supplementary material for: Transcriptomic Divergence and Associated Markers Between Genomic Lineages of Silver Catfish ( Rhamdia quelen )
Source: Ecol Evol. 2025 Mar 13;15(3):e71021. doi: 10.1002/ece3.71021 (PMC11904098; doi:10.1002/ece3.71021)
Supplement: Supplementary file 1 — Data S1. [file ECE3-15-e71021-s009.docx]

**Supplemental file I.** Samples, locality, mitochondrial lineage (Rq4 and Rq6), and sex (M: Male; F: Female; I: Indeterminate) of the specimens included in the RNA pools.

| Individuals | Locality | Mitochondrial lineages | Genbank  Accession Number | Sex | Brain | Head Kidney | Liver | Skeletal muscle | Ovary | Testes |
| --- | --- | --- | --- | --- | --- | --- | --- | --- | --- | --- |
| P2301 | Río Arapey | *Rq4* | PP898343 | F | B-N | K-N | L-N | M-N |  |  |
| P2302 | Río Arapey | *Rq4* | PP898344 | F |  | K-N | L-N | M-N |  |  |
| P2303 | Río Arapey | *Rq4* | PP898345 | F | B-N | K-N | L-N | M-N |  |  |
| P2304 | Río Arapey | *Rq4* | PP898346 | F | B-N | K-N | L-N | M-N | OVA |  |
| P2305 | Río Arapey | *Rq4* | PP898347 | M | B-N | K-N | L-N | M-N |  |  |
| P2306 | Río Arapey | *Rq4* | PP898348 | F | B-N |  | L-N | M-N |  |  |
| P2307 | Río Arapey | *Rq4* | PP898349 | F | B-N |  | L-N | M-N |  |  |
| P2308 | Río Arapey | *Rq4* | PP898350 | F | B-N | K-N | L-N | M-N |  |  |
| P2309 | Río Arapey | *Rq4* | PP898351 | F | B-N | K-N | L-N | M-N | OVA |  |
| P2310 | Río Arapey | *Rq4* | PP898352 | F |  | K-N | L-N |  |  |  |
| P2311 | Río Arapey | *Rq4* | PP898353 | F | B-N | K-N |  | M-N |  |  |
| P2312 | Río Arapey | *Rq6* | PP898354 | I |  |  |  |  |  |  |
| P2313 | Río Arapey | *Rq4* | PP898355 | F | B-N | K-N |  |  |  |  |
| P2315 | Laguna Blanca | *Rq6* | PP898356 | F | B-S | K-S | L-S | M-S |  |  |
| P2316 | Laguna Blanca | *Rq6* | PP898357 | F | B-S | K-S | L-S | M-S |  |  |
| P2317 | Laguna Blanca | *Rq6* | PP898358 | M | B-S | K-S | L-S | M-S |  | TES |
| P2318 | Laguna Blanca | *Rq6* | PP898359 | F | B-S | K-S | L-S | M-S | OVA |  |
| P2319 | Laguna Blanca | *Rq6* | PP898360 | F | B-S | K-S | L-S | M-S |  |  |
| P2320 | Laguna Blanca | *Rq6* | PP898361 | F | B-S | K-S | L-S | M-S |  |  |
| P2321 | Laguna Blanca | *Rq6* | PP898362 | M | B-S | K-S | L-S | M-S |  | TES |
| P2322 | Laguna Blanca | *Rq6* | PP898363 | F | B-S | K-S | L-S | M-S |  |  |
| P2323 | Laguna Blanca | *Rq6* | PP898364 | F | B-S | K-S | L-S | M-S | OVA |  |
| P2324 | Laguna Blanca | *Rq6* | PP898365 | F | B-S | K-S | L-S | M-S |  |  |

B-N (brain pool of the North lineage), B-S (brain pool of the South lineage), K-N (head kidney pool of the North lineage), K-S (head kidney pool of the South lineage), L-N (liver pool of the North lineage), L-S (liver pool of the South lineage), M-N (skeletal muscle pool of the North lineage), M-S (skeletal muscle pool of the South lineage), OVA (ovary pool) and TES (testis pool of the North lineage) indicate samples used in each pool.
