# Supplementary material for: Transcriptomic Divergence and Associated Markers Between Genomic Lineages of Silver Catfish ( Rhamdia quelen )
Source: Ecol Evol. 2025 Mar 13;15(3):e71021. doi: 10.1002/ece3.71021 (PMC11904098; doi:10.1002/ece3.71021)
Supplement: Supplementary file 2 — Data S2. [file ECE3-15-e71021-s004.docx]

**Supplemental File II.** Summary of sequencing, assembly and mapping of transcriptome data of the 10 *R. quelen* libraries

| **Sequencing statistics** | **B-N** | **B-S** | **K-N** | **K-S** | **L-N** | **L-S** | **M-N** | **M-S** | **OVA** | **TES** | **Total** |
| --- | --- | --- | --- | --- | --- | --- | --- | --- | --- | --- | --- |
| RIN | 7.5 | 5.6 | 7.4 | 8.5 | 8.4 | 8.0 | 6.2 | 7.9 | 5.5 | 9.8 |  |
| Raw paired reads | 34845811 | 33401650 | 42677954 | 34712779 | 31505963 | 37394945 | 33830673 | 33193509 | 33027993 | 31011721 | 345602998 |
| Filtered reads | 1916264 | 1856054 | 2431056 | 1874064 | 1699255 | 1913565 | 1709183 | 1820012 | 1755311 | 1894341 | 18869105 |
| %GC content | 45 | 46 | 47 | 48 | 47 | 47 | 49 | 49 | 48 | 47 | 48 |
| Paired reads passed | 32929547 | 31545596 | 40246898 | 32838715 | 29806708 | 35481380 | 32121490 | 31373497 | 31272682 | 29117380 | 326733893 |
| % Both paired reads passed | 94.50 | 94.44 | 94.30 | 94.60 | 94.61 | 94.88 | 94.95 | 94.52 | 94.69 | 93.89 | 94.54 |
| Total surved (Gb) | 6.44 | 6.17 | 7.90 | 6.45 | 5.87 | 7.00 | 6.31 | 6.17 | 6.15 | 5.70 | 64.17 |
| Q20 surved (%) | 99.51 | 99.52 | 99.52 | 99.54 | 99.53 | 99.59 | 99.50 | 99.53 | 99.55 | 99.48 | 99.53 |
| Q30 surved (%) | 97.92 | 97.94 | 97.91 | 97.99 | 97.92 | 98.16 | 97.94 | 97.92 | 98.02 | 97.85 | 97.96 |
| Annotation statistics |  |  |  |  |  |  |  |  |  |  | 24433 |
| Consistent expressed annotated genes TPM ≥ 5 | 10866 | 12141 | 7950 | 7522 | 4929 | 5276 | 7868 | 5156 | 7455 | 10,463 |  |
| Expressed annotated genes (TPM ≥ 1) | 16988 | 17853 | 13118 | 12373 | 10344 | 10897 | 12378 | 11966 | 11941 | 17161 |  |
| Average gene length (bp) | 2100 | 2065 | 2026 | 2068 | 2084 | 2095 | 2223 | 2102 | 2181 | 2015 | 1780 |
| Gene length range | 201-30789 | 201-30789 | 201-30789 | 201-30789 | 201-21946 | 201-21946 | 201-19529 | 201-19529 | 201-17546 | 201-30789 | 201-30789 |
| Number of expressed genes < 500pb | 2947 | 3211 | 2357 | 2109 | 1704 | 1773 | 1698 | 2026 | 1647 | 3133 | 5695 |
| Number of expressed genes ≥ 500pb | 14051 | 14642 | 10761 | 10264 | 8641 | 9125 | 10681 | 9971 | 10295 | 14029 | 18738 |
| Genes expressed with annotated GO terms | 6596 | 6871 | 5083 | 4879 | 4289 | 4458 | 5148 | 4959 | 5040 | 6631 | 8917 |

Q20, Q30: PHRED quality scores.
